# *Bacillus cereus* eﬄux protein BC3310 – a multidrug transporter of the unknown major facilitator family, UMF-2

**DOI:** 10.3389/fmicb.2015.01063

**Published:** 2015-10-12

**Authors:** Jasmin K. Kroeger, Karl Hassan, Aniko Vörös, Roger Simm, Massoud Saidijam, Kim E. Bettaney, Andreas Bechthold, Ian T. Paulsen, Peter J. F. Henderson, Anne-Brit Kolstø

**Affiliations:** ^1^Laboratory for Microbial Dynamics, Department of Pharmaceutical Biosciences, School of Pharmacy, University of OsloOslo, Norway; ^2^Institut für Pharmazeutische Biologie und Biotechnologie, Albert-Ludwigs UniversitätFreiburg, Germany; ^3^Department of Chemistry and Biomolecular Sciences, Macquarie University, SydneyNSW, Australia; ^4^School of BioMedical Sciences and Astbury Centre for Structural Molecular Biology, University of LeedsLeeds, UK

**Keywords:** MFS, drug resistance, eﬄux protein, *Bacillus cereus*, UMF-2

## Abstract

Phylogenetic classification divides the major facilitator superfamily (MFS) into 82 families, including 25 families that are comprised of transporters with no characterized functions. This study describes functional data for BC3310 from *Bacillus cereus* ATCC 14579, a member of the “unknown major facilitator family-2” (UMF-2). BC3310 was shown to be a multidrug eﬄux pump conferring resistance to ethidium bromide, SDS and silver nitrate when heterologously expressed in *Escherichia coli* DH5α *ΔacrAB*. A conserved aspartate residue (D105) in putative transmembrane helix 4 was identified, which was essential for the energy dependent ethidium bromide eﬄux by BC3310. Transport proteins of the MFS comprise specific sequence motifs. Sequence analysis of UMF-2 proteins revealed that they carry a variant of the MFS motif A, which may be used as a marker to distinguish easily between this family and other MFS proteins. Genes orthologous to *bc3310* are highly conserved within the *B. cereus* group of organisms and thus belong to the core genome, suggesting an important conserved functional role in the normal physiology of these bacteria.

## Introduction

*Bacillus cereus sensu stricto* (*B. cereus*) is a Gram-positive, endospore forming organism known to cause foodborne illness in humans. It is a member of the *B. cereus* group of bacteria (*Bacillus cereus sensu lato*) that, in addition to *B. cereus* encompasses the species *B. anthracis, B. thuringiensis, B. mycoides, B. pseudomycoides, B. weihenstephanensis*, and *B. cytotoxicus* (Kolsto et al., 2009; Guinebretiere et al., 2013). The *B. cereus* group members are genetically closely related with high level of syntheny (conserved gene order). The high similarity results in an intertwinement of the *B. cereus, B. thuringiensis*, and *B. weihenstephanensis* branches in the phylogenetic tree (Ash et al., 1991). However, the *B. cereus* group organisms exhibit different phenotypes, inhabit diverse ecological niches and are pathogenic against different hosts. The three species *B. mycoides, B. pseudomycoides*, and *B. weihenstephanansis* are regarded as non-pathogenic. *B. anthracis* is the causative agent of anthrax in humans and animals (Mock and Fouet, 2001). *B. thuringiensis* is an insect pathogen that is commercially used as a biopesticide (Melo et al., 2014). *B. cytotoxicus* causes enteritis in humans and is thermotolerant and highly cytotoxic (Guinebretiere et al., 2013). In the natural environment *B. cereus* is found as a saprophyte in soil, associated with the rizosphere of plants and in the gut of invertebrates (Jensen et al., 2003; Berg et al., 2005). Even though *B. cereus* is most frequently associated with food-borne enteric infections in humans, it is able to cause other local or systemic infections such as endophthalmitis, cutaneous infections, endocarditis, central nervous system infection, or bacteremia (Steen et al., 1992; Callegan et al., 1999; Centers for Disease Control and Prevention, 2005; Callegan et al., 2006; Martinez et al., 2007; Kim et al., 2010; Sasahara et al., 2011; Stevens et al., 2012). Clinically serious infections of *B. cereus* are treated with antibiotics such as carbapenems, clindamycin, ciprofloxacin, and vancomycin (Kervick et al., 1990; Bottone, 2010; Uchino et al., 2012; Matsuda et al., 2014). However, resistance against carbapenem and clindamycin has been reported, which eventually led to failed treatments including cases with fatal outcomes (Kervick et al., 1990; Kiyomizu et al., 2008; Savini et al., 2009; Uchino et al., 2012).

According to the transportdb database, the *B. cereus* group strains constitute between 390 and 455 transporters per strain (Ren et al., 2007; Ren and Paulsen, 2007). The unusually high number of transporters per *B. cereus* group strain may reflect the different lifestyles of these bacteria. Importantly, each group member contains approximately 100 transporters, predicted to eﬄux drugs.

Drug eﬄux systems are part of the resistance machinery to counteract antibiotics (Sun et al., 2014). They are divided into six different transporter superfamilies: (i) MFS (major facilitator superfamily); (ii) ABC (ATP binding cassette) transporter superfamily; (iii) MATE (multidrug and toxic compound extrusion) family; (iv) RND (resistance nodulation division) family; (v) DMT (drug/metabolite transporter) superfamily, and (vi) PACE (proteobacterial antimicrobial compound eﬄux) (Poole, 2007; Hassan et al., 2015). Of these, MFS pumps constitute the majority of eﬄux transporters encoded in *B. cereus* group strains, typically more than 50 per strain. The MFS comprises secondary transporters that use the electrochemical gradient of protons or sodium ions across the cell membrane to energize substrate transport, including drug eﬄux (Pao et al., 1998; Saier et al., 1999; Reddy et al., 2012). The ‘transporter classification system’ (see http://www.tcdb.org/) classifies the MFS into 82 families. With respect to drug eﬄux pumps, the drug:H^+^ antiporter families (DHA)1 to 3 are the largest and best investigated drug exporter families in the MFS (Saier et al., 2014).

In this study, we characterize the phylogenetic and some functional properties of the putative multidrug transporter BC3310 from *B. cereus* ATCC 14579. BC3310 was classified by *in silico* analysis as a member of the major facilitator superfamily and the phylogenetic relationship within this group was determined. A deletion mutant of *bc3310* was constructed and overexpression of BC3310 allowed for functional characterization in a heterogenous host as well as purification and partial biochemical characterization *in vitro*.

## Materials and Methods

### Bioinformatics Analyses

Bacterial sequence information was collected using the IMG homepage from the Joint Genome Institute (Markowitz et al., 2012). Sequence alignments were performed using MEGA MUSCLE alignment with default settings (Tamura et al., 2013) and the phylogenetic tree was constructed using MrBayes (Ronquist et al., 2012). Prediction of the transmembrane helices was done by submitting the primary protein sequence of BC3310 (UniProt Q81B77) to HMMTOP (Tusnady and Simon, 2001).

### Construction of *B. cereus bc3310* Deletion Mutant

A markerless mutant of *bc3310* was constructed as described (Simm et al., 2012) in the *B. cereus* ATCC 14579 wild type according to the method of Janes and Stibitz (2006) and using the primers listed in **Table 1**. The *B. cereus* plasmid pBClin15 was lost during the process of making the markerless mutant and therefore a plasmid cured strain was used for phenotypic comparison as in previous investigations (Voros et al., 2013). The presence of the deletion was confirmed by sequencing. *B. cereus* was grown in LB medium at 30°C, unless otherwise stated.

**Table 1:** Primers used in this study.

Primer for	Sequence (5′→3′)
**Overexpression in pTTQ18**	
pTTQ18-bc3310F	CATGGATCCATGCGTTTTACTTTTTGGATTATGG
pTTQ18-bc3310R	CCGCCTGCAGCGGTTGTTTTGTCATGCCC
**D105 mutants**	
bc3310_D105N_f	GATTTCTAGTTGGAGTTGGAAATCATATGCTTCATGTCGGAAC
bc3310_D105N_r	GTTCCGACATGAAGCATATGATTTCCAACTCCAACTAGAAATC
bc3310_D105A_f	GATTTCTAGTTGGAGTTGGAGCTCATATGCTTCATGTCGGAAC
bc3310_D105A_r	GTTCCGACATGAAGCATATGAGCTCCAACTCCAACTAGAAATC
bc3310_D105E_f	GATTTCTAGTTGGAGTTGGAGAACATATGCTTCATGTCGGAAC
bc3310_D105E_r	GTTCCGACATGAAGCATATGTTCTCCAACTCCAACTAGAAATC
**Deletion mutant**	
dbc3310_5′_f	CGCGGATCCATGAACAAACTATATTAC
dbc3310_5′_r	CAATTTCCCTTCCCAAAAAGTAAAACGCAT
dbc3310_3′_f	GTTTTACTTTTTGGGAAGGGAAATTGAAGTAA
dbc3310_3′_r	ACGCGTCGACTAGTTTGATATACCTGTTC

### *Escherichia coli* BC3310 Expression Constructs

The expression levels of genes cloned into pTTQ18-based plasmids are inducible by isopropyl β-D-thiogalactopyranoside (IPTG). Furthermore, the genes are fused with a sequence coding for a C-terminal (His)_6_ tag for identification and purification of the expressed protein. The plasmid construct pTTQ18-bc3310 (pbc3310) was made by general molecular biology techniques according to Sambrook and Russell (2001) by amplifying the gene *bc3310* from genomic DNA of *B. cereus* ATCC 14579 using the primers listed in **Table 1**. The plasmids for expressing BC3310 D105 mutants pbc3310D105A, pbc3310D105N, and pbc3310D105E were made using sequence and ligation-independent cloning (Li and Elledge, 2007). The presence of each mutation was confirmed by sequencing. The *E. coli* strain DH5α Δ*acrAB* (Simm et al., 2012) carrying pTTQ18 empty vector or the overexpression plasmids was made for minimal inhibition concentration (MIC) testing. For protein purification the *E. coli* strain BL21 was transformed with pbc3310.

*Escherichia coli* strains harboring plasmids were grown in 50 or 250 ml LB medium with ampicillin (100 μg ml^-1^) at 37°C and 180 rpm in 250 ml or 1 l baﬄed flasks or on LB agar plates at 37°C, unless otherwise stated.

### MIC Tests

Overnight cultures of *B. cereus* ATCC 14579 (without pBClin) and *B. cereus Δ3310* or *E. coli* DH5α *ΔacrAB* (Simm et al., 2012) with relevant plasmid were inoculated 1:100 and grown to an OD_600_ between 0.8 and 1.0 at 37°C and 180 rpm. These pre-cultures were diluted to a final OD_600_ of 0.02. The test was performed at least three times in duplicate in microtiter plates and antibiotics were added in a 2-fold serial dilution. For susceptibility assay using *E. coli* strains 100 μg ml^-1^ ampicillin and 0.01 mM IPTG were added to all cultures. The cultures were incubated at 37°C for 20–24 h and visually inspected for growth. The lowest concentration, at which no growth was observed, was determined as the MIC.

### Ethidium Bromide Accumulation Assay

*Escherichia coli* strains DH5α *ΔacrAB* with the plasmids pTTQ18 and pbc3310 were grown on LB agar plates supplemented with 100 μg ml^-1^ ampicillin and 0.01 mM IPTG at 37°C over night. Cells were collected with a loop and resuspended in PBS supplemented with 0.4% glucose (pH 7-7.4) to an OD_600_ of 1.000 (±0.005). These cells were applied on a microtiter plate and, where appropriate, carbonyl cyanide *m*-chlorophenylhydrazone (CCCP) was added to achieve an end concentration of 200 μM. Thereafter, ethidium bromide was added to an end concentration of 25 μM and the fluorescence change was measured over 60 min in a Safire spectrophotometer (Tecan, Crailsheim, Germany) with excitation and emission wavelength of 518 and 605 nm, respectively. Duplicate measurements were recorded on at least two cultures.

### Heterologous Expression of BC3310 and Its Mutants with (His)_6_-tag and Western Blot

Overnight cultures of *E. coli* DH5α *ΔacrAB* carrying pbc3310, the empty vector (pTTQ18) or plasmids encoding the *bc3310* mutants (pbc3310D105A, pbc3310D105N, or pbc3310D105E) were transferred to fresh LB (amp) medium and grown to an OD_680_ between 0.4 and 0.6. Expression was induced with 0.75 mM IPTG and the cultures were grown for another 3 h. For quantification of expression, Western blot assays were performed. One milliliter of the overexpression cultures was harvested by centrifugation at 15000 *g*, 4°C for 5 min. The pellet was washed (20 mM Tris-HCl pH 7.6, 100 mM NaCl, 5% glycerol, 1 mM phenylmethanesulfonylfluoride (PMSF)) and resuspended depending on cell mass in ice-cold lysis buffer (50 mM Tris-HCl pH 7.6, 100 mM NaCl, 5% glycerol, 5 mM β-mercaptoethanol, 1 mM PMSF, 1 μg ml^-1^ DNase). Cells were lysed by continuous sonication for 25 min in a cold water bath. SDS-PAGE and Western blots were performed as described in Sambrook and Russell (2001). (His)_6_-tag detection was done using a mouse anti-(His)_6_ antibody (Qiagen, Hilden, Germany) and a horse anti-mouse horseradish peroxidase-labeled secondary antibody (New England Biolabs. ECL advanced chemiluminescence detection reagent (Amersham Pharmacia Biotech, Pittsburgh, PA, USA) was used and chemiluminescence was measured by using the Analyzer Universal hood (Bio Rad, München) and the Quantity one 4.6.6 Software. Quantification was performed by pixel counting of five biological replicates on five different Western blots.

### Purification of the BC3310 Protein by Affinity Chromatography

For protein expression and purification, the method described by Ward et al. (2000) was used. In short, *E. coli* strain BL21 pbc3310 was grown in 2TY medium (1.6% tryptone, 1% yeast extract, 0.5% sodium chloride, pH 7) and expression was induced at an OD_680_ between 0.4 and 0.6 with 0.75 mM IPTG. The culture was grown for another 3 h and cells were harvested. For inner membrane preparation, *E. coli* cells were resuspended in 20 mM Tris-HCl (pH 8.0), 0.5 mM EDTA and kept frozen at –80°C. After thawing, cells were disrupted with a Continuous Flow Disruptor (Constant Systems, UK) and inner membranes isolated by sucrose gradient centrifugation. Samples were kept at –80°C in Tris-HCl (pH 7.5) and EDTA.

Inner membranes were solubilized in 20 mM CAPSO (pH 10.0), 300 mM sodium chloride, 20% glycerol, 1% *n*-dodecyl β-D-maltoside (DDM), 20 mM imidazole (pH10.0). Immobilized metal affinity chromatography (IMAC) was performed using 20 mM CAPSO (pH 10.0), 10% glycerol, 0.05% DDM, 20 mM imidazole (pH 10.0) as wash buffer and 20 mM CAPSO (pH 10.0), 200 mM imidazole, 5% glycerol, and 0.05% DDM as elution buffer.

### Circular Dichroism Measurement

Purified protein was washed using a spin concentrator with 20 mM CAPSO (pH 10.0), 5% glycerol and 0.05% DDM until imidazole-free. CD spectral analysis was performed from 270 to 195 nm in a 1 nm step resolution using a spectropolarimeter (Jasco J-715) with constant nitrogen flushing and a scan rate of 10 nm min^-1^. Response time was set at 1 s with a sensitivity of 100 mdeg and 10 nm bandwidth. The data comprised an accumulation of 20 scans, from which the buffer contribution was subtracted.

## Results

### BC3310 is Conserved in the *B. cereus* Group

**FIGURE 1:**
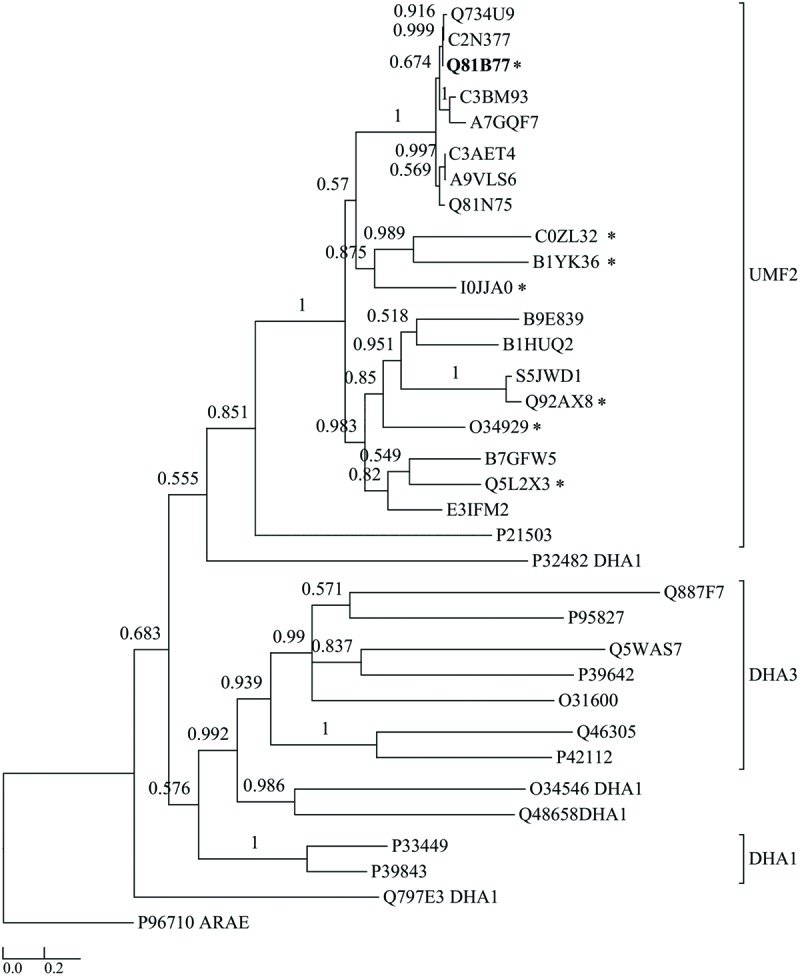
**Dendrogram comparing BC3310 from *Bacillus cereus* ATCC 14579 with orthologous proteins and other multidrug transporters from the DHA1 and DHA3 families.** BC3310 from *B. cereus* ATCC 14579 (UniProt accession number: Q81B77; bold font) and orthologous proteins from *B. cereus* ATCC 10987 (Q734U9), *B. cereus* ATCC 10876 (C2N377), *B. anthracis* str. Ames (Q81N75), *B. cereus* ssp. cytotoxis (A7GQF7), *B. weihenstephanensis* (A9VLS6), *B. mycoides* (C3AET4), *B. pseudomycoides* (C3BM93), *Geobacillus* sp. Y4.1MC1 (E3IFM2), *Halobacillus halophilus* (I0JJA0), *B. subtilis* (O34929), *Listeria innocua* (Q92AX8), *Listeria monocytogenes* (S5JWD1), *Geobacillus kaustophilus* (Q5L2X3), *Lysinibacillus sphaericus* (B1HUQ2), *Exiguobacterium sibiricum* (B1YK36), *Anoxybacillus flavithermus* (B7GFW5), *M. caseolyticus* (B9E839), *Brevibacillus brevis* (C0ZL32), *Escherichia coli* (P21503), and DHA1 proteins from *Lactococcus lactis* (Q48658), *B. subtilis* (Q797E3, O34546, P39843, P33449), *Pseudomonas aeruginosa* (P32482) and DHA3 proteins from *Streptococcus pyrogenes* (P95827), *B. subtilis* (P39642, O31600, P42112), *B. clausii* (Q5WAS7), *Pseudomonas syringae* (Q887F7), *Clostridium perfringens* (Q46305) and the sugar transporter AraE from *B. subtilis* (P96710) as an outgroup were used to build the tree. Posterior probability values are shown at each node and the bar represents the expected number of amino acid substitutions per site. The seven protein sequences marked with ^∗^ were aligned in **Figure 5**.

To date, 228 strains of the *B. cereus* group of bacteria have been sequenced (Markowitz et al., 2012). A BLASTP search showed that the protein BC3310 is highly conserved within this group. In 225 strains BC3310 orthologs with >91% amino acid identity were identified. The predicted ortholog from the reduced genome sized *B. cereus cytotoxis* NVH 391-98 displayed 88% identity. The two strains (*B. anthracis* 3154 and *B. anthracis* A2012) in which no BC3310 ortholog was found are draft genomes which display a gap at the relevant genomic position (data not shown). Orthologs of the BC3310 protein are also found in other bacteria of the order Bacillales including *B. subtilis* (51% amino acid identity), *Listeria innocua* (47% amino acid identity), *Geobacillus kaustophilus* (47% amino acid identity), *Lysinibacillus sphaericus* (50% amino acid identity), *Exiguobacterium sibiricum* (39% amino acid identity), *Anoxybacillus flavithermus* (49% amino acid identity), *Macrococcus caseolyticus* (42% amino acid identity), *Brevibacillus brevis* (41% amino acid identity). The phylogenetic relationship of BC3310 to a selection of orthologs is depicted in a dendrogram (**Figure 1**). BC3310 clusters very closely with orthologous proteins from other *B. cereus* group members, thus forming a distinct cluster separate from the orthologs of other Bacillales species.

### *B. cereus* Δ*bc3310* is More Susceptible to Ethidium Bromide Compared to the Wild Type

To examine the role of BC3310 in conferring drug tolerance in *B. cereus* ATCC 14579 a microbroth dilution test was conducted comparing the *B. cereus* wild type to its isogenic markerless knock-out mutant. Growth of the strains in twofold serial dilutions of ten compounds, including antibiotics from different classes, was tested. The susceptibility of the *Δbc3310* mutant only differed from the susceptibility of the wild type strain for one of the 10 tested compounds. *B. cereus Δbc3310* was two times more susceptible to ethidium bromide compared to the wild type (**Table 2**). It is possible that redundancy among eﬄux transporters masks the substrate range of the BC3310 transporter or that the transporter is not expressed under the conditions studied. Hence, a heterologous *E. coli* expression system with a hypersensitive *E. coli* strain and IPTG-inducible BC3310 expression was used to further investigate possible substrates.

**Table 2:** Minimal inhibition concentration (MIC) of *E. coli* DH5α *ΔacrAB* expressing BC3310 (pbc3310) compared to empty vector control (pTTQ18) and *Bacillus cereus* ATCC 14579 *Δbc3310* (Δbc3310) compared to *B. cereus* ATCC 14579 (wild type).

	MIC [μg ml^-1^]						
	*E. coli* DH5α *ΔacrAB*				*B. cereus* ATCC 14579		
Compound	Empty vector	pbc3310	§		Wild type	Δ*bc3310*	§
Apramycin	n.d.^∗^	n.d.			12.5	12.5	1
Chloramphenicol	1.25	1.25	1		3.13	3.13	1
Erythromycin	12.5	12.5	1		0.25	0.25	1
Kanamycin	2.5	2.5	1		12.5	12.5	1
Lincomycin	400	400	1		n.d.	n.d.	
Nalidixic acid	n.d.	n.d.			5	5	1
Novobiocin	1.25	1.25	1		n.d.	n.d.	
Phleomycin	n.d.	n.d.			50	50	1
Tetracycline	1.25	1.25	1		1.25	1.25	1
Ethidium bromide	3.13	12.5	4		50	25	0.5
SDS	100	400	4		100	100	1
Silver nitrate	1.3	2.7	2		0.43	0.43	1

*§represents fold difference between *E. coli* or *B. cereus* strains, experiments were conducted at least three times in duplicate.*

*^∗^ denotes not determined.*

### Expression of BC3310 Protein in *E. coli*

**FIGURE 2:**
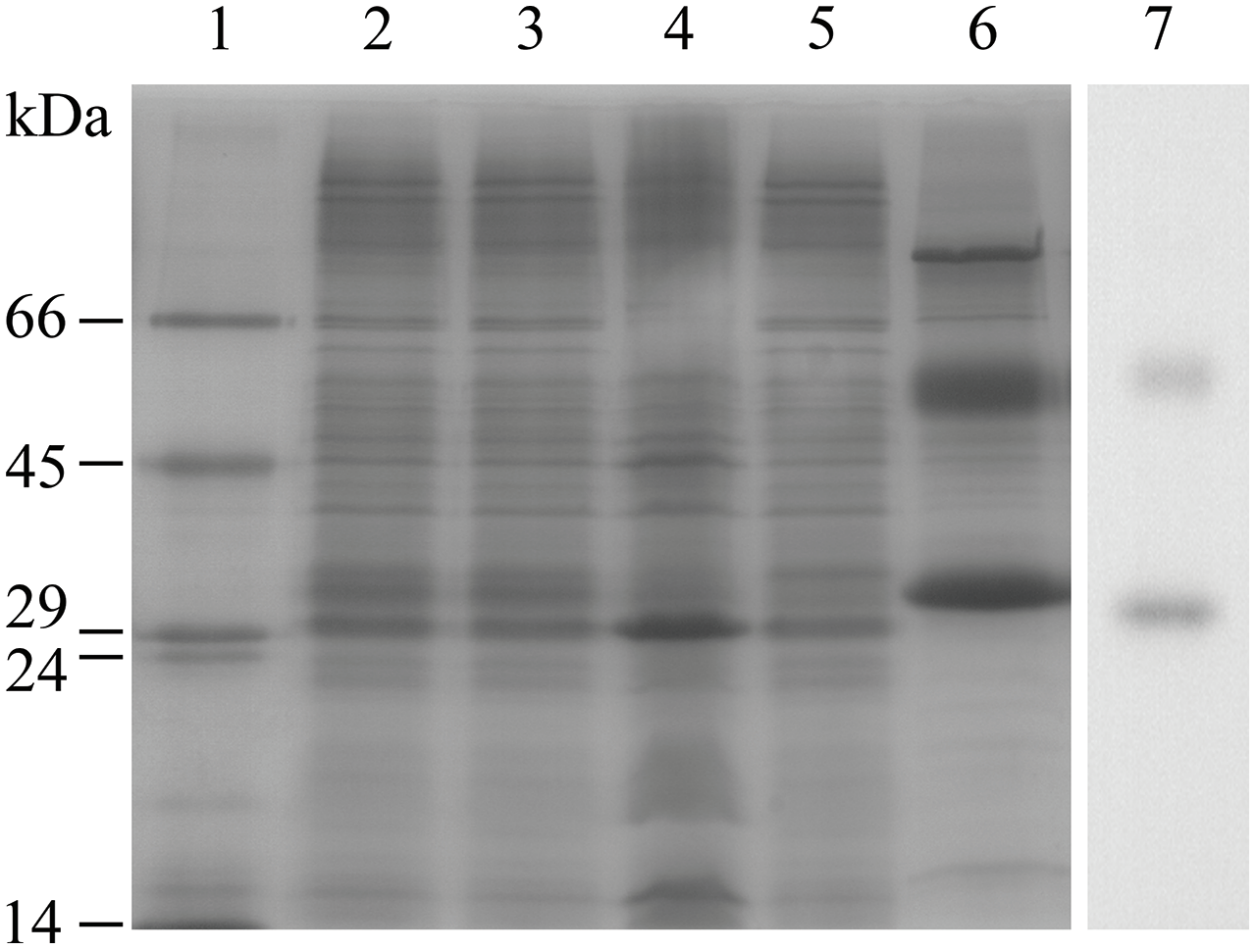
**Purification of BC3310-RGSHis_6_.** BC3310 was expressed with RGS(His)_6_-tag in *E. coli* and inner and outer membranes were separated. BC3310-RGSHis_6_ was solubilized with DDM and purified by immobilized metal affinity chromatography (IMAC). The SDS-PAGE was loaded as follows and stained with Coomassie Blue: (1) molecular weight marker; (2) inner membrane fraction; (3) solubilized protein; (4) unsolubilized protein; (5) flow-through after IMAC binding; (6) eluted protein. (7) Western blot detection of eluted BC3310 protein after affinity chromatography using an antibody to the RGSHis_6_ epitope.

**FIGURE 3:**
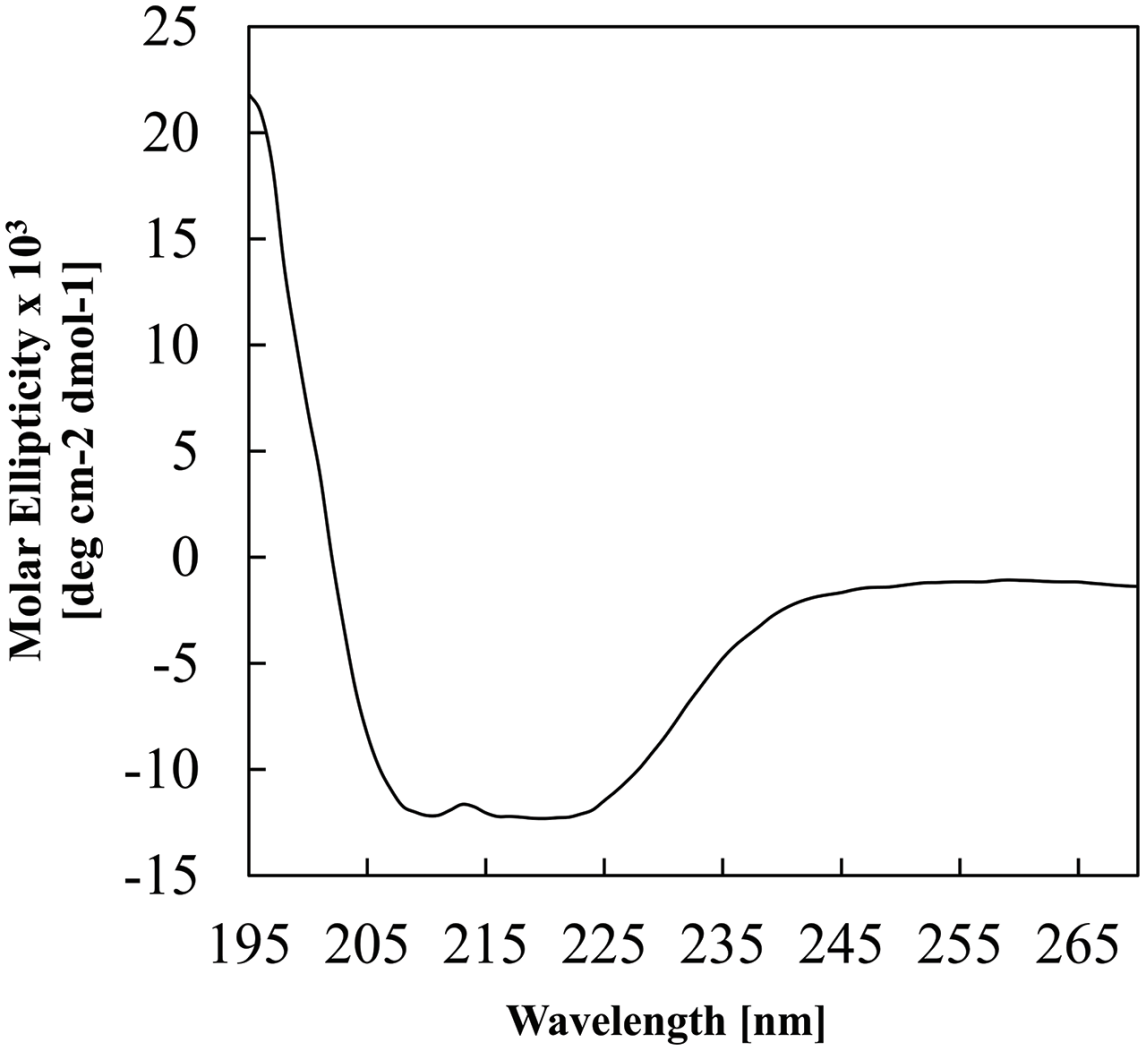
**Circular dichroism analysis of purified BC3310-RGSHis_6_ protein.** The analysis was performed at 1 nm intervals over 270–190 nm with a scan rate of 10 nm min^-1^. The spectrum represents an averaged accumulation of 20 scans, from which the buffer contribution was subtracted.

The ability of *E. coli* to heterologously express intact BC3310 protein was investigated. The *bc3310* gene was cloned into the expression vector pTTQ18 as described (Saidijam et al., 2006, 2011; Szakonyi et al., 2007). BC3310 was expressed with a C-terminal RGSHis_6_ tag and detected by Western blotting using an antibody against the RGSHis_6_ tag (**Figure 2**). The protein was solubilized from the inner membrane fraction with DDMand purified by affinity chromatography (**Figure 2**). The major band on the Coomassie stained gel was subjected to Edman degradation and confirmed to contain the first eight predicted amino acids of BC3310. Topology analysis with HMMTOP predicted 12 transmembrane helices in the BC3310 transport protein. Circular dichroism measurements of the purified protein resulted in a spectrum with nodes at 210 and 222 nm (**Figure 3**), indicating a prevailing α-helical structure (Wallace et al., 2003) and thus confirming the integrity of the heterologously produced protein.

Thereafter the substrate range of heterologously expressed BC3310 was determined. A susceptibility assay was performed using *E. coli* DH5α *ΔacrAB* in which the major multidrug eﬄux complex was disrupted. The MICs of different compounds for the strain expressing BC3310 from pTTQ18 were compared to the MICs for the empty vector control. The *E. coli* strain expressing BC3310 showed a fourfold higher MIC for ethidium bromide and SDS and a twofold higher MIC for silver nitrate (**Table 2**).

### Ethidium Bromide Eﬄux of BC3310 is Disrupted by CCCP

**FIGURE 4:**
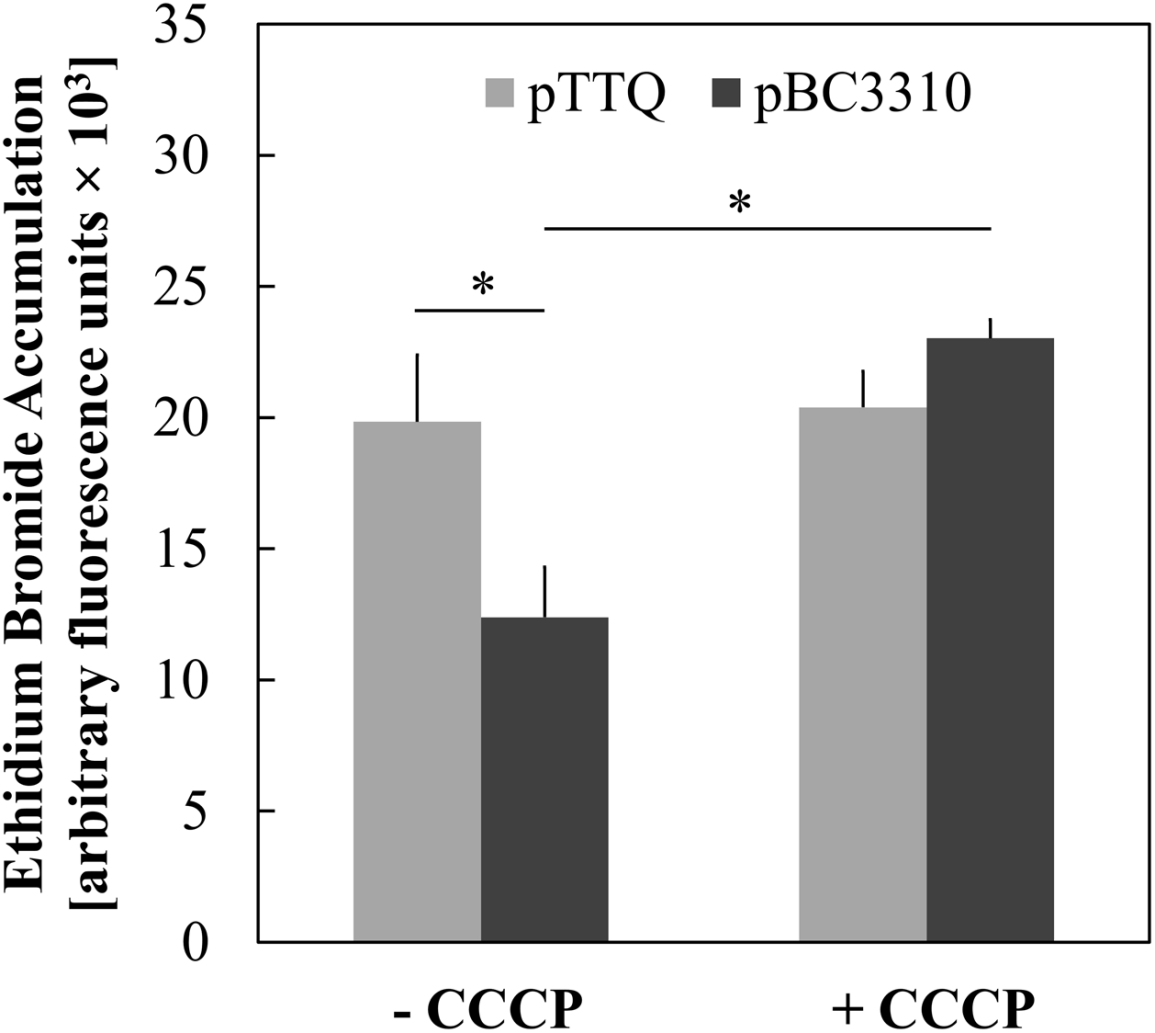
**Uncoupler-sensitive eﬄux of ethidium associated with expression of BC3310.** Accumulation of ethidium bromide after 30 min was measured in IPTG-induced *E. coli* DH5α Δ*acrAB* host cells either expressing *bc3310* (pBC3310, dark gray) or not using an empty vector control (pTTQ18, light gray) without (-CCCP) and with addition of CCCP (+ CCCP). Values are means of four independent experiments and error bars indicate standard deviations, ^∗^*p* < 0.01, unpaired Student’s *t*-test.

Major facilitator superfamily eﬄux proteins are secondary active transporters that utilize the electrochemical gradient across the cell membrane to extrude compounds. The BC3310 protein sequence displays motifs characteristic of an MFS transporter (see below) and so the ability of BC3310 to confer resistance to ethidium bromide by means of drug eﬄux was investigated further. A whole cell ethidium bromide accumulation assay with the *E. coli* DH5α *ΔacrAB* strain expressing BC3310 was performed. Ethidium bromide fluoresces upon binding to double-stranded DNA, and the fluorescence intensity correlates with the accumulation of ethidium bromide. The *E. coli* strain expressing *bc3310* (pbc3310) showed less fluorescence compared to the empty vector control (pTTQ18), thereby implying that BC3310 exports ethidium bromide (**Figure 4**). Addition of the protonophore CCCP led to an increase in fluorescence intensity in the strain expressing *bc3310* to approximately the control level (pTTQ18) (**Figure 4**, dark gray bars). This increase indicates the inability of BC3310 to export ethidium bromide due to the disruption of the electrochemical gradient.

### Mutation of the Conserved Aspartic Acid Residue (D105) Abolishes Ethidium Bromide Eﬄux

**FIGURE 5:**
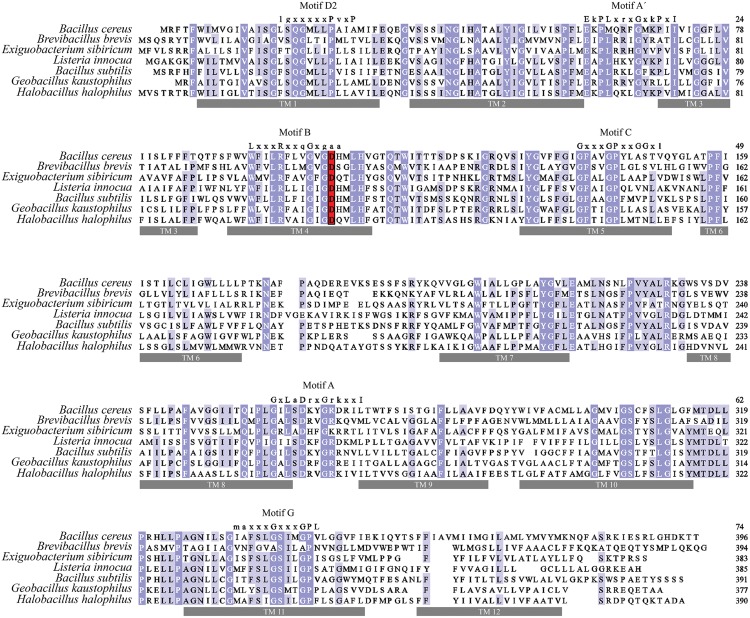
**Multiple sequence alignment of BC3310 and its homologs.** BC3310 from *B. cereus* ATCC 14579 (UniProt accession number: Q81B77) was aligned using MUSCLE with orthologs from *Brevibacillus brevis* (C0ZL32), *Exiguobacterium sibiricum* (B1YK36), *Listeria innocua* (Q92AX8), *B. subtilis* (O34929), *Geobacillus kaustophilus* (Q5L2X3) and *Halobacillus halophilus* (I0JJA0). Shading corresponds to >80% (dark blue), >60% (blue), >40% (light blue), and ≤40% (white) amino acid identity, respectively. The conserved acidic residue in transmembrane region 4 is displayed in red. Transmembrane regions have gray bars under and conserved MFS motifs are depicted above the sequence.

**FIGURE 6:**
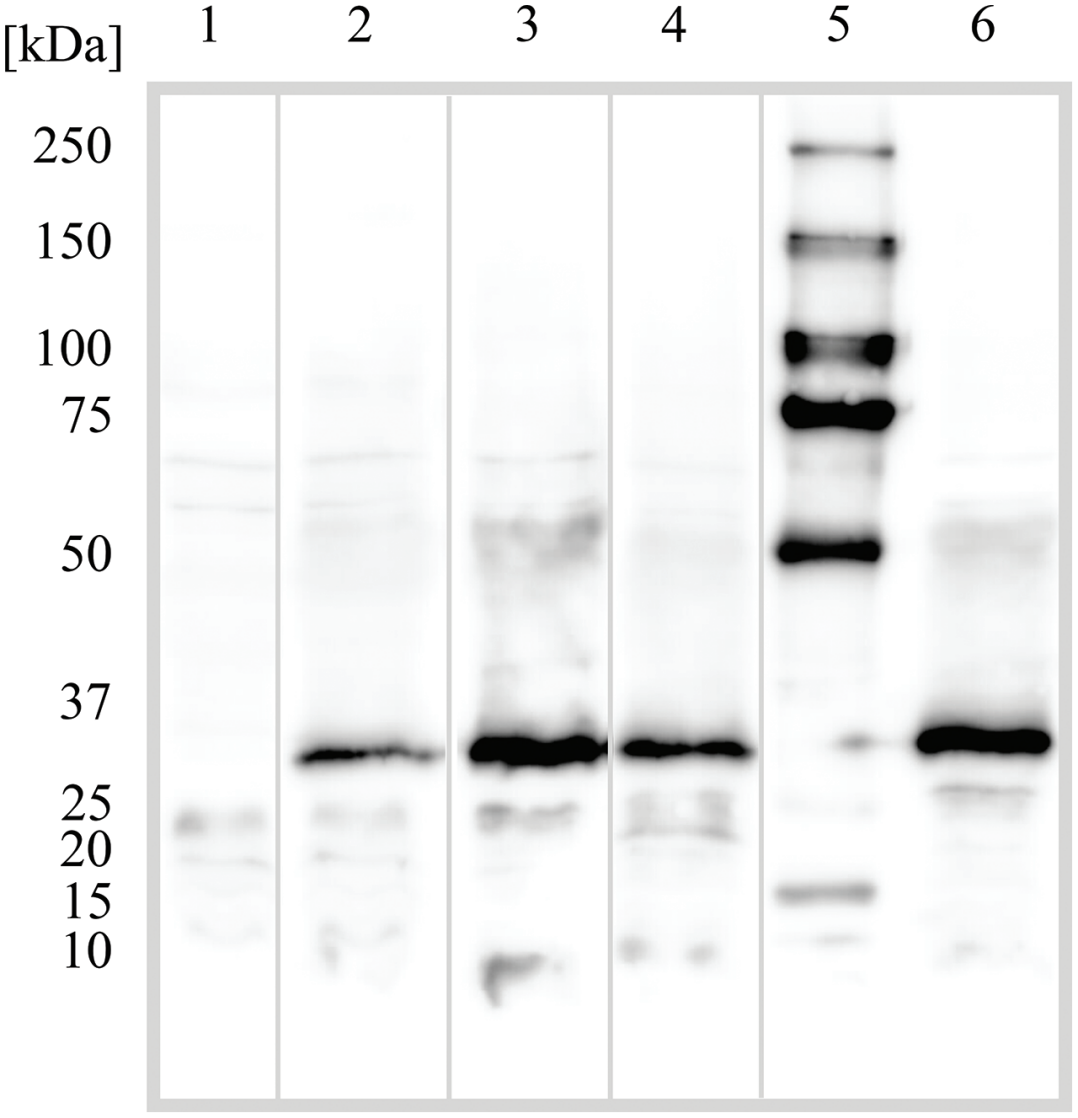
**Western blot detection of the BC3310 protein and its mutants in *Escherichia coli*.** *E. coli* DH5α *ΔacrAB* was transformed with empty pTTQ18 vector plasmid or pTTQ18 with the indicated insert; wild type BC3310 with his-tag and mutants D105N, D105A, and D105E under an IPTG inducible promoter. The cells were grown and prepared for SDS-PAGE as described in Section “Materials and Methods”. Numbers indicate: (1) empty pTTQ18 vector; (2) BC3310 wild type; (3) D105N mutant; (4) D105A mutant; (5) molecular weight marker; (6) D105E mutant. BC3310 and its mutants migrate at ∼30 kDa.

Proton or substrate translocations by transport proteins often require acidic residues within transmembrane helices (Paulsen et al., 1996a; Edgar and Bibi, 1997; Sanderson et al., 1998; Dang et al., 2010). Sequence alignment of BC3310 with orthologous proteins revealed a conserved acidic residue in putative TMS 4 (**Figure 5**). In order to investigate the importance of this conserved aspartate residue (D105) for eﬄux activity, mutational analyses were conducted. Three constructs were made in which the aspartate residue was mutated to glutamate (D105E), asparagine (D105N), or alanine (D105A). The expression of the mutant proteins was detected and quantified by Western blot (**Figure 6**). This showed that the expression of all mutant proteins was three to four times higher compared to the expression of wild type protein. MIC determination of ethidium bromide and silver nitrate was performed to investigate the functionality of the mutant BC3310 proteins (**Table 3**). Even though more mutant protein was expressed, the susceptibility of strains expressing mutant BC3310 was reduced to levels approximating those of the empty vector control-strain. Thus, mutational change of the aspartate residue to another acidic or a structurally similar residue abolished the eﬄux ability of BC3310 for ethidium bromide and silver nitrate, indicating that both the size and charge of the side chain at position 105 are important for protein function.

**Table 3:** Relative expression rate and relative MIC of *E. coli* strains producing no BC3310, BC3310 wild type, D105N, D105A, or D105E mutant protein.

*E. coli* DH5α *ΔacrAB* producing	Relative expression^b^ [%]	Relative resistance to [%]^a^	
		Ethidium bromide	Silver nitrate
BC3310 wild-type	100	100	100
No BC3310	NA^c^	20	60
D105N	440	20	50
D105A	330	25	50
D105E	380	30	60

*^a^MICs were determined in *E. coli* DH5α Δ*acrAB* pTTQ18 expressing *bc3310* in LB media or LB media without NaCl (for silver nitrate) supplemented with 0,04 mM IPTG and 75 *μ*g ml^*-1*^ carbenicillin.*

*^b^average of five different Western blots of five different cultures.*

*^c^NA, not applicable.*

### BC3310 Belongs to the UMF-2 Family of the MFS

BC3310 showed prevailing α-helical structure in our CD analysis and is predicted to be a 12-TMS multidrug transporter belonging to the MFS. Most of the 12 TMS-containing MFS proteins that eﬄux several drugs are members of the drug:H^+^ antiporter families DHA1 and DHA3. To determine if BC3310 belongs to one of these families within the MFS, a multiple alignment of sequences orthologous to BC3310 and sequences from the well described DHA1 and DHA3 families was performed. From this alignment a dendrogram was built which showed clustering of BC3310 and orthologs in a distinct clade separate from the DHA1 and DHA3 family proteins included in the analysis (**Figure 1**). This analysis supported the transporter classification database (TCDB) division of YfkF, the BC3310 ortholog in *B. subtilis*, into a separate family, the unknown major facilitator family-2 (UMF-2) (Saier et al., 2014).

### Transport Proteins within the UMF-2 Family Contain a Variant of the MFS Signature Motif A

Sequence alignment revealed that amino acid sequence motifs characteristic for MFS transporters, namely motif A, B, C, and G were conserved in BC3310 and orthologous proteins (**Figure 5**) (Henderson and Maiden, 1987; Griffith et al., 1992; Paulsen et al., 1996b). Motif A is conserved in the loop region between transmembrane segments (TMS) 2 and 3, and has been called the MFS signature motif due to its conservation across the superfamily. In the majority of MFS transporters, including the DHA1 family proteins, the motif A consensus sequence is G-x-L-a-D-r/k-x-G-r/k-r/k-x-x-I (x indicating any amino acid; capital and lower case letters representing amino acid frequency of >70% and 40–70%, respectively; Henderson and Maiden, 1987; Griffith et al., 1992; Paulsen et al., 1996b). However, a functional variant of this motif has been described in the *Clostridium perfringens* DHA3 family tetracycline eﬄux protein TetA(P): E-x-P-x-x-x-x-x-**D**-x-x-x-**R**-**K** (bold letters overlap with D, r/k,r/k of the canonical motif A) (Bannam et al., 2004). In BC3310 and its orthologs a modified motif A (motif A′) was identified, which represents a hybrid of the canonical motif A and the TetA(P) motif A (**Table 4**) (Paulsen et al., 1996b; Bannam et al., 2004). The N-terminal sequence of motif A′ in BC3310 orthologs resembles the TetA(P) (DHA3) motif A, with E and P conserved in both motifs, whereas the C-terminal sequence corresponds to the DHA1 motif A. This results in the BC3310 modified motif A′ sequence **E**-r/k-**P**-L-x-**r/k**-x-**G**-x-**r/k**-P-x-**I** (bold letters correspond to sequences of the previously described motif A sequences).

**Table 4:** Consensus sequences of motif A variants found in MFS drug export families.

MFS family	Consensus sequence of motif A variants					
DHA1					G	x	L	a	D	**r/k**	x	**G**	r/k	**r/k**	x	x	**I**
TetA(P) (DHA3)	**E**	x	**P**	x	x	x	x	x	D	x	x	x	R	K			
BC3310 (UMF-2)	**E**	r/k	**P**	–	–	–	–	L	x	**r/k**	x	**G**	x	**r/k**	P	x	**I**

*x indicates any amino acid; capital, and lower case letters represent an amino acid frequency occurrence of >70 and 40–70%, respectively; bold letters indicate an overlap with conserved amino acids of the DHA1 or DHA3 family.*

As in other MFS transporters, a second motif A-like sequence is present between TMS 8 and TMS 9 in BC3310 (consensus sequence: **G**-x-**L**-S-**D**-**r/k**-x-**G**-**R**-**r/k**-x-x-**i**/l). This sequence coincides more with the signature motif A compared to the motif A′ sequence between TMS 2 and TMS 3 (Henderson and Maiden, 1987; Griffith et al., 1992).

## Discussion

Heterologous expression of BC3310 in a drug hypersusceptible *E. coli* strain increased the tolerance of the bacteria to AgNO_3_, SDS, and ethidium bromide, indicating that it has a role in resistance to multiple drugs. Whole cell accumulation assays of ethidium bromide in *E. coli* expressing *bc3310* demonstrated CCCP-sensitive eﬄux of ethidium in the drug hypersusceptible *E. coli* strain confirming a function as a drug eﬄux protein. Hence, BC3310 is an energy-dependent multidrug eﬄux pump. Inactivation of *bc3310* in *B. cereus* ATCC 14579, also resulted in increased susceptibility to ethidium bromide, but not to SDS or AgNO_3_, suggesting, low basal expression of *bc3310* under the conditions used in our experiments. It has, however, previously been reported that addition of 1 mM AgNO_3_ to exponentially growing cultures of *B. cereus* ATCC 14579 induced expression of *bc3310* (Babu et al., 2011) and we detected AgNO_3_-induced temporal expression of *bc3310* by qRT-PCR under our experimental settings (data not shown). Therefore, although BC3310 seems to have a role in transport of Ag^+^ and/or ${\mathrm{NO}}_{3}^{–}$ it is not essential in conferring AgNO_3_ resistance under the conditions tested, but may be important under specific circumstances. *B. cereus* ATCC 14579 contains 93 genes annotated as drug transporter which corresponds to 1.7% of the protein coding genes in the genome (Saidijam et al., 2006, 2011; Ren et al., 2007). In comparison, *B. subtilis* and *E. coli* display 32 and 37 genes encoding drug transport proteins, respectively, which correspond to 0.8 and 0.9% of the protein coding genes (Nishino and Yamaguchi, 2001; Ren et al., 2004). Considering the high number of annotated drug transporter genes in the genome of *B. cereus*, it is possible that one or more transporters compensate for the loss of BC3310, thereby concealing a potential effect of a gene disruption.

The eﬄux of ethidium bromide by BC3310 is dependent on a conserved aspartate residue, which could not be replaced by another acidic or hydrophobic amino acid. This indicates an important role of the aspartate residue at position 105 (D105) in the putative TMS 4. This residue is also conserved in BC3310 orthologs. Even though this aspartate residue is not reported to be one of the conserved residues, it falls into the boundaries of motif B. The motif B sequence of BC3310 and orthologs is W-**x**-**x**-L-**R**-**x**-**x**-x-**G**-**x**-**G**-D-x which overlaps to a large degree with canonical motif B L-**x**-**x**-x-**R**-**x**-**x**-q-**G**-**x**-**g**-a-a (bold letters indicate matching amino acids, underlined letter is D105 in BC3310). Motif B contains an absolutely conserved basic amino acid residue which is proposed to play a role in proton transfer (Paulsen and Skurray, 1993). This residue is also conserved in BC3310 (R98).

Sequence analyses classified BC3310 into the UMF-2 family of the MFS which is distinct from the well characterized drug eﬄux families DHA1 and DHA3 and consists of previously uncharacterized proteins. We have thus described the first functional data for a member of the UMF-2 family and showed that it includes multidrug eﬄux proteins. Previously transporters belonging to (at least) five of the 82 different families have been implicated in multidrug eﬄux. Besides the mentioned DHA1 and DHA3 families with 12 TMS-containing transporters, multidrug eﬄux proteins have been described for the Organic Cation Transporter family (2.A.1.19) (Koepsell, 2013). In addition, the DHA2 family is known to contain multidrug eﬄux proteins with 14 TMS (Paulsen et al., 1996b) and the gene encoding MdrA in *Streptomyces coelicolor*, classified into the Acriflavin Sensitivity family (2.A.1.36), is regulated by a TetR repressor that recognizes multiple drugs (Hayashi et al., 2013).

Interestingly, BC3310 and its orthologs contain an alternative motif A′ consensus sequence E-r/k-P-L-x-r/k-x-G-x-r/k-P-x-I between putative TMS 2 and 3. We propose that this consensus sequence can be used as a marker to distinguish the UMF-2 family from other MFS families. The presence of a second motif A in BC3310 is likely due to the duplication of 6 TMS during the evolution of the 12-TMS MFS transporters (Paulsen and Skurray, 1993). Similarly, motif G relates to a duplication of motif C (antiporter motif) (Paulsen et al., 1996b). Motif C is only conserved in exporters and not in importers (Paulsen and Skurray, 1993). This motif is also found with a high similarity (including the functionally important GP dipeptide; De Jesus et al., 2005) in BC3310 and orthologs which is in line with the eﬄux function of BC3310. Little similarity to MFS motif D2 is observed in the sequence alignment of BC3310 orthologs. As reported previously, motif D2 does not appear to be highly conserved in recently investigated 12-TMS MFS transporters and a function has not yet been assigned (Paulsen et al., 1996b; Kapoor et al., 2009).

The gene encoding the BC3310 transporter is highly conserved in the genomes of the *B. cereus* group members indicating that *bc3310* belongs to the core genome of the *B. cereus* group. Comparison of the *bc3310* genomic region of *B. cereus* ATCC 14579 with the equivalent regions of selected *B. cereus* group members, *B. cereus* ATCC 10987, *B. cereus* ATCC 10876, *B. anthracis* Ames Ancestor A2084, *B. thuringiensis* sv. kurstaki YBT-1520, and *B. mycoides* ATCC 6462 showed the same gene organization. The different species of the *B. cereus* group inhabit many different niches and display a high number of eﬄux transporter genes in the genome compared to other bacteria which could account for the different lifestyles (Saidijam et al., 2006, 2011). Thus, genes conserved in the genomes of the *B. cereus* group might play a role in the fundamental maintenance of physiological functions. Preliminary phenotypic microarray data using BIOLOG, however, did not reveal significant differences between *B. cereus* ATCC 14579 wild type and Δ*bc3310* mutant. Condition-dependent transcriptome analyses of the *bc3310* ortholog, *yfkF*, in *B. subtilis* revealed relatively constant transcriptional activity across the conditions investigated (Nicolas et al., 2012). The highest level of gene expression was observed in cells within stationary (OD_600_ ∼2) or transition (OD_600_ ∼1.4) growth phases in LB medium or LB medium supplemented with glucose as well as on LB agar. Ethanol stress conditions revealed the lowest expression of this gene. Furthermore *yfkF* is predicted to be under the control of the housekeeping sigma factor SigA (Nicolas et al., 2012). Transcription of genes encoding multidrug transporters with a major role in protecting the cell against toxic compounds is generally activated by transcription factors that recognize toxic compounds or stress signals, such as AcrR, SoxS, MarR, and Rob in the case of AcrAB of *E. coli* (Ma et al., 1996; Sulavik et al., 2001; Randall and Woodward, 2002; Rosenberg et al., 2003). This fact and the minor intrinsic susceptibility against toxic compounds in the *B. cereus Δbc3310* deletion mutant indicate that BC3310 is not a potent multidrug transporter with a main role in protecting the cell against toxic xenobiotics. It rather hints to an ancient and maybe general function in the normal physiology of the *B. cereus* group of bacteria. To further elucidate the role of this transporter the inactivation of other eﬄux proteins might be required.

Taken together, we have performed the first phylogenetic and functional characterization of a member of the UMF-2. The amino acid sequence of BC3310 comprises known motifs of the 12-TMS MFS transporters with a modified motif A′ between TMS 2 and TMS 3. BC3310 is a multidrug transporter with confirmed predominant α-helical structure. It confers resistance to ethidium bromide, SDS, and silver nitrate when expressed in *E. coli*. The export of ethidium bromide is energy dependent and requires a conserved aspartate residue in TMS 4. The deletion of *bc3310* in *B. cereus* resulted in increased susceptibility to ethidium bromide under the conditions tested. The high conservation of *bc3310* within the *B. cereus* group genomes indicates that it is part of the core genome. We hypothesize that the intrinsic role of BC3310 is not as a typical multidrug transporter, but rather as an important component in the normal physiology of the bacteria, under conditions that still remain to be identified.

## Conflict of Interest Statement

The authors declare that the research was conducted in the absence of any commercial or financial relationships that could be construed as a potential conflict of interest.
